# Intermittent concurrent use of clopidogrel and proton pump inhibitors did not increase risk of adverse clinical outcomes in Chinese patients with coronary artery disease

**DOI:** 10.1186/s12872-021-01884-z

**Published:** 2021-02-05

**Authors:** Wanbing He, Xiaorong Shu, Enyi Zhu, Bingqing Deng, Yongqing Lin, Xiaoying Wu, Zenan Zhou, Jingfeng Wang, Ruqiong Nie

**Affiliations:** Department of Cardiology, Sun Yat-Sen Memorial Hospital of Sun Yat-Sen University, 107 Yanjiang West Rd., Guangzhou, 510120 Guangdong Province China

**Keywords:** Clopidogrel, Proton pump inhibitor, Coronary heart disease, Gastrointestinal bleeding, NACE, Clinical outcomes

## Abstract

**Background:**

Proton pump inhibitors (PPIs) are frequently prescribed to patients with coronary heart disease (CHD) under antiplatelet therapy to prevent gastrointestinal (GI) bleeding. However, its clinical impact is still under debate, especially in Asian population. This study was undertaken to explore the effects of concurrent use of clopidogrel and PPIs on the clinical outcomes in Chinese patients with CHD in secondary prevention.

**Methods:**

A single-center retrospective study was conducted in 638 patients with CHD on consecutive clopidogrel therapy for at least 1 year. After 18-month follow-up, adverse clinical events were collected. Cox regression was used to calculate hazard ratios (HR) and 95% confidence interval (CI) for the effect of PPI use on the outcomes. A total of 638 patients were recruited from 2014 to 2015 in this study, among whom 201 were sustained PPI users, 188 were intermittent PPI users and the remaining 249 were non-PPI users.

**Results:**

Compared with sustained PPI users, intermittent use of PPIs was associated with a lower risk of stroke, major adverse cardiac events (MACE) and net adverse clinical event (NACE) (stroke: adjusted HR: 0.109, 95% CI 0.014–0.878, *p* = 0.037; MACE: adjusted HR: 0.293, 95% CI 0.119–0.722; *p* = 0.008; NACE: adjusted HR: 0.357, 95% CI 0.162–0.786, *p* = 0.011). Subgroup analysis further revealed the benefit of intermittent PPI use was significant in male CHD patients over 60 years old, with hypertension or chronic kidney disease, and undergoing percutaneous coronary intervention during hospitalization.

**Conclusion:**

The current findings suggest that the intermittent concurrent use of PPIs and clopidogrel is not associated with an increased risk of 18-month adverse clinical outcomes, and intermittent use of PPIs is associated with a lower rate of MACE and NACE.

## Background

Cardiovascular diseases (CVD) have become the leading causes of death worldwide. According to the 2013 Global Burden of Disease Study, approximately 17 million people died of CVD worldwide, accounting for approximately 31.5% of the total number of deaths [1]. Among the different types of CVDs, the mortality of coronary artery disease (CHD) was the highest [2, 3]. Therefore, it is important to prevent CHD.

The rupture or erosion of vulnerable plaques that induces platelet activation and aggregation are the key pathophysiological mechanisms, which contributes to the deaths of patients with CHD. Therefore, antiplatelet therapy is critical to reduce cardiovascular deaths in patients with CHD [4]. Clopidogrel combined with aspirin, called dual antiplatelet therapy (DAPT), is the basic treatment strategy for CHD, especially for acute coronary syndrome (ACS). DAPT can effectively prevent percutaneous coronary intervention (PCI) from in-stent thrombosis and reduce cardiovascular adverse events. Nevertheless, the risks of gastrointestinal (GI) and other bleeding events are significantly increased after treatment with DAPT. Thus, patients who accept DAPT, especially after PCI, often take proton pump inhibitors (PPIs) at the same time. Most PPIs such as omeprazole, esomeprazole and lansoprazole, are mainly metabolized by liver cytochrome (CYP) P450 isoenzyme CYP2C19, which also regulates clopidogrel metabolism. When combined with clopidogrel, PPIs may exert a competitive inhibitory effect, and thus affect the therapeutic effects of clopidogrel. Previous pharmacodynamic studies showed that the concurrent use of PPIs could reduce anti-platelet activities of clopidogrel [5–8]. However, the results from clinical trials were inconsistent. Some large clinical studies such as ADAPT-DES, BASKET, and CAPRIE indicated that combined use was associated with an increased risk of cardiovascular adverse events [4, 9, 10]. However, the results from other large-scale prospective clinical studies such as COGENT, CREDO, PRINCIPLE-TIMI 44, PRODIGY, TRITON-TIMI 38 showed the opposite results, which indicated that the concurrent use of PPIs and clopidogrel did not increase the risk of net adverse clinical events (NACE) and even had benefits by reducing the risk of GI bleeding [7, 9, 11, 12]. Furthermore, clopidogrel was more frequently used in combination with PPIs in Asian countries than that in Western countries [13, 14]. However, researches on the clinical effect of the concurrent use on both cardiovascular adverse events and GI bleeding in the Asian population are very limited. Therefore, this study was aimed to investigate the effect of concurrent use of clopidogrel and PPIs on clinical outcomes (composite endpoint events such as cardiovascular events and GI bleeding) in Chinese patients with CHD.

## Materials and methods

### Study population

This was a single-center retrospective study of consecutive CHD patients referred to the Sun Yat-sen Memorial Hospital of Sun Yat-sen University between January 2014 and April 2015. Patients over 18 years old with diagnosed CHD and under clopidogrel (75 mg/d) treatment over 12 months were included. Patients who met one of the following criteria were excluded: (1) anemia (hemoglobin < 90 g/L); (2) platelet count < 100 × 10^9^/L, or with hematological diseases, which affect the number or function of platelets; (3) severe liver insufficiency with alanine transaminase (ALT) or glutamic-oxaloacetic transaminase (AST) three times higher than normal level; (4) under long-term non-steroidal anti-inflammatory drugs (NSAIDS) (except aspirin) or glucocorticoid treatment; (5) transferred to other hospital for treatment other than death, or the information for drug use can not be collected.

The follow-up study was conducted at 12 and 18 months via outpatient follow-up or telephone follow-up. The follow-up content included all-cause deaths, cardiovascular events, gastrointestinal side effects, gastrointestinal bleeding, chest pain and other discomfort symptoms. The consumption of drugs (clopidogrel and PPIs) during the follow-up was obtained via the outpatient prescription system. In addition, the reasons for those patients being not in the hospital for continuous hospitalization were also collected via telephone follow-up.

The study protocol conformed to the ethical guidelines of the 1975 Declaration of Helsinki as reflected in a priori approval by the Ethics Committee of Sun Yat-sen Memorial Hospital of Sun Yat-sen University. Informed consent was obtained from each participant.

### Baseline clinical data

In this study, the hospitalization information was collected by querying the medical record system of the hospital. If the patient had multiple hospitalization records, only the most relevant and the earliest record within the enrolling period were chosen as baseline data. The following data were collected: (1) Demographic data: age, sex, body mass index (BMI), blood pressure (BP), smoking and alcohol status; (2) Comorbidity: CHD, acute myocardial infarction (AMI), chronic heart failure (CHF), hypertension, hyperlipidemia, atrial fibrillation (AF), transient ischemic attack (TIA), stroke, diabetes mellitus (DM), chronic kidney disease (CKD), gastroduodenal ulcer, gastrointestinal hemorrhage et al. (3) Surgical history: PCI, coronary artery bypass graft (CABG), cardiac device implantation, or other surgical history.

### Laboratory parameters

Laboratory parameters were all measured by using blood sample. Each patient were fasted overnight for at least 10 h before venipuncture. Platelets counts, serum creatinine, fasting plasma glucose (FPG), total cholesterol (TC), high-density lipoprotein cholesterol (HDL-C), low-density lipoprotein cholesterol (LDL-C), triglyceride (TG), high-sensitivity C-reactive protein (hs-CRP) and creatine phosphokinase isoenzyme (CK-MB) were analyzed by a standardized and certified TBA-120 auto-analyzer (Toshiba Medical Systems, Japan) in the institutional central laboratory. The CYP2C19 genotype was also detected. According to the current international classification, different CYP2C19 genotypes can be classified into four metabolic types: (1) extensive (normal) metabolizers (EMs), carrying two normal alleles (such as *1/*1); (2) intermediate metabolizers (IMs), carrying one loss-of-function (LOF) allele (e.g. *1/*2); (3) poor metabolizers (PMs), carrying two LOF alleles (e.g. *2/*2, *2/*3, *3/*3); (4) ultra-rapid metabolizers (UMs), carrying one or two gain-of-function (GOF) allele (such as *1/*17, *17/*17) [15]. The frequency of GOF allele in Chinese population is very low (about 1/1000), and thus this type of genotype was not detected in this study [16].

### Drug use

The PPIs regimens were determined by the clinicians according to the actual condition of the patient and were not affected by this study. The dosage and frequency of PPIs were recorded in each patient’s visit. According to the length of time of taking PPIs, this study divided the patients into three groups: (1) Sustained PPI users: use of PPIs during the follow-up period more than 30 days; (2) Intermittent PPI users: ever use of PPIs during follow-up period but less than 30 days; (3) non-PPI users: no PPIs were taken during the follow-up period. Use of other drugs such as aspirin, angiotensin converting enzyme inhibitor (ACEI), angiotensin II receptor antagonist (ARB), statins, β-blockers, calcium channel blocker (CCB) were also collected according to the records of discharge from hospital. All included patients were under a standardized treatment of CHD secondary prevention determined by the clinicians blinded to this study.

### Clinical outcomes

Clinical outcomes included all-cause death, re-hospitalization, myocardial infarction (MI), target vessel revascularization, stroke (ischemic), GI events or other bleeding events. GI events include: (1) symptoms of GI upset and diagnosis of GI diseases including gastritis or duodenitis, gastric or duodenal ulcer, gastric or duodenal obstruction, gastric or duodenal perforation; (2) GI bleeding: symptoms of hematemesis, melena, positive fecal occult blood, or the appearance of active bleeding under gastroscopy; (3) gastroesophageal reflux disease (GERD) determined by gastroscopy. Other bleeding events were defined as types 2, 3, and 5 in the 2011 Bleeding Academic Research Consortium (BARC) bleeding standard [17]. Major adverse cardiac event (MACE) was defined as the sum of cardiac deaths, MI, target vessel revascularization and stroke. Net adverse clinical event (NACE) was defined as the sum of all-cause deaths, MI, target vessel revascularization, stroke, GI bleeding, and BARC type 2, 3, and 5 events.

### Statistical analysis

Data were presented as frequencies for categorical variables, mean values with standard deviation (SD) for normally distributed continuous variables and median values with 25th and 75th percentiles for ordinal variables. The comparison of continuous variables among multiple groups was performed by one-way analysis of variance (ANOVA) or Kruskal–Wallis test; while Chi-square test was performed for the comparison of categorical variables. The univariate Kaplan–Meier method was used to analyze the survival free from the clinical events, and the overall survival rate was compared using log-rank test. Cox proportional hazard regression model was used to adjust all possible confounding factors, which might influence the outcome of clinical events. In this study, the confounding factors included in the model were based on the differences among three groups and the factors associated with the occurrence of clinical events. The final factors included in the adjusted model were BMI, history of MI, stroke, DM, gastroduodenal ulcer, and alcohol consumption, whether to take β-blockers, aspirin, hemoglobin, whether to be hospitalized PCI. The final results were expressed as adjusted hazard ratio (adjusted HR) and its 95% confidence interval (95% CI). In order to further analyze the factors that affect the occurrence of clinical events, sensitivity analysis on the occurrence of NACE was also conducted. Subgroups were defined as age (≤ 6 0 years old and > 60 years old), gender (male, female), and hypertension, stroke, DM, CKD, gastroduodenal ulcer, type of PPIs (pantoprazole and other types), hospitalization for PCI, and CYP2C19 genotypes (EM, IM, PM) and compared by using the adjusted Cox proportional hazard regression model mentioned above. All statistical analyses were performed using SPSS 22.0 statistical software (SPSS, Inc, Chicago, IL). *p* < 0.05 was considered as statistical significance.

## Results

### Baseline characteristics of patients included

**Fig. 1:**
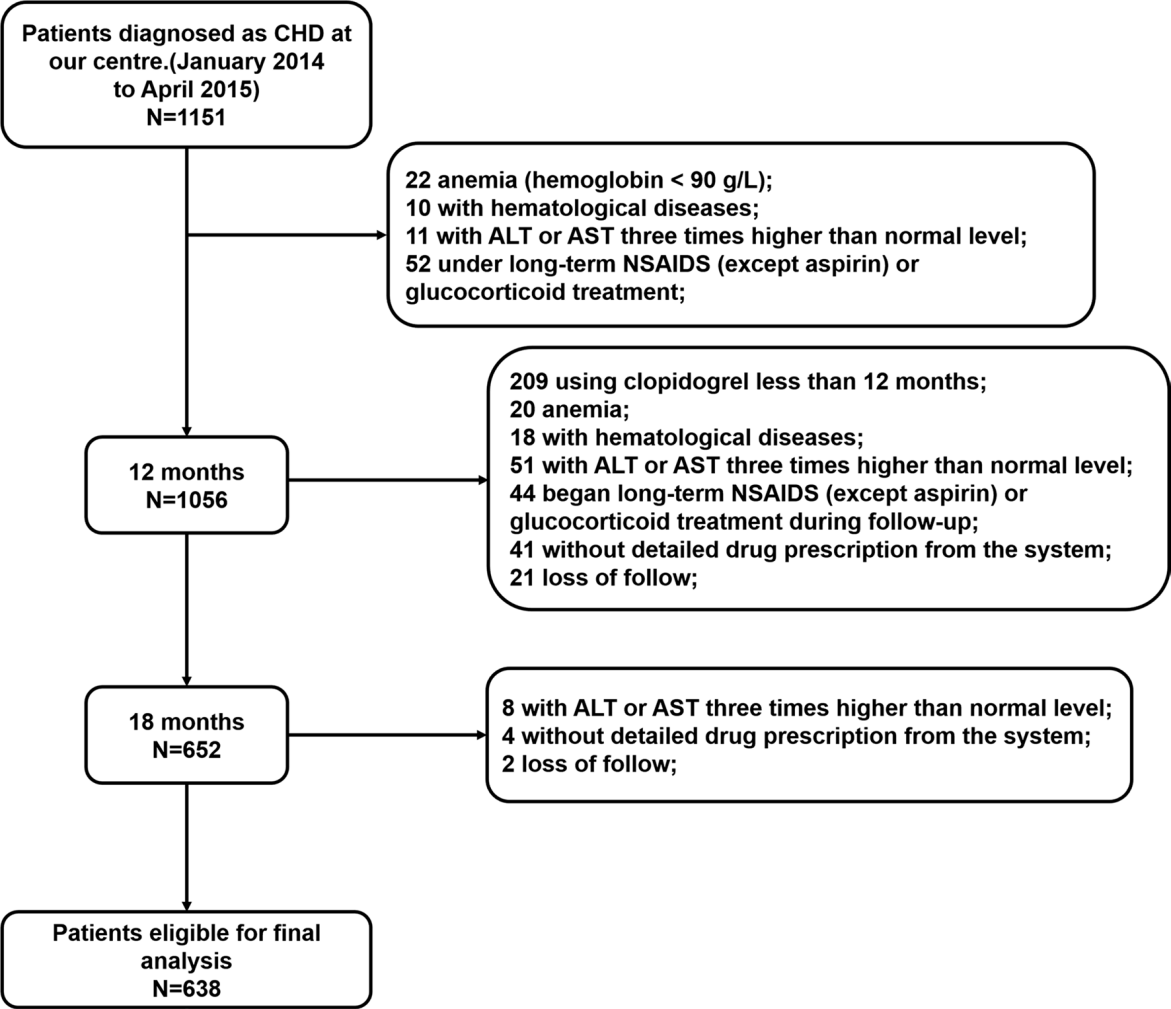
Flow chart for patient selection process

**Fig. 2:**
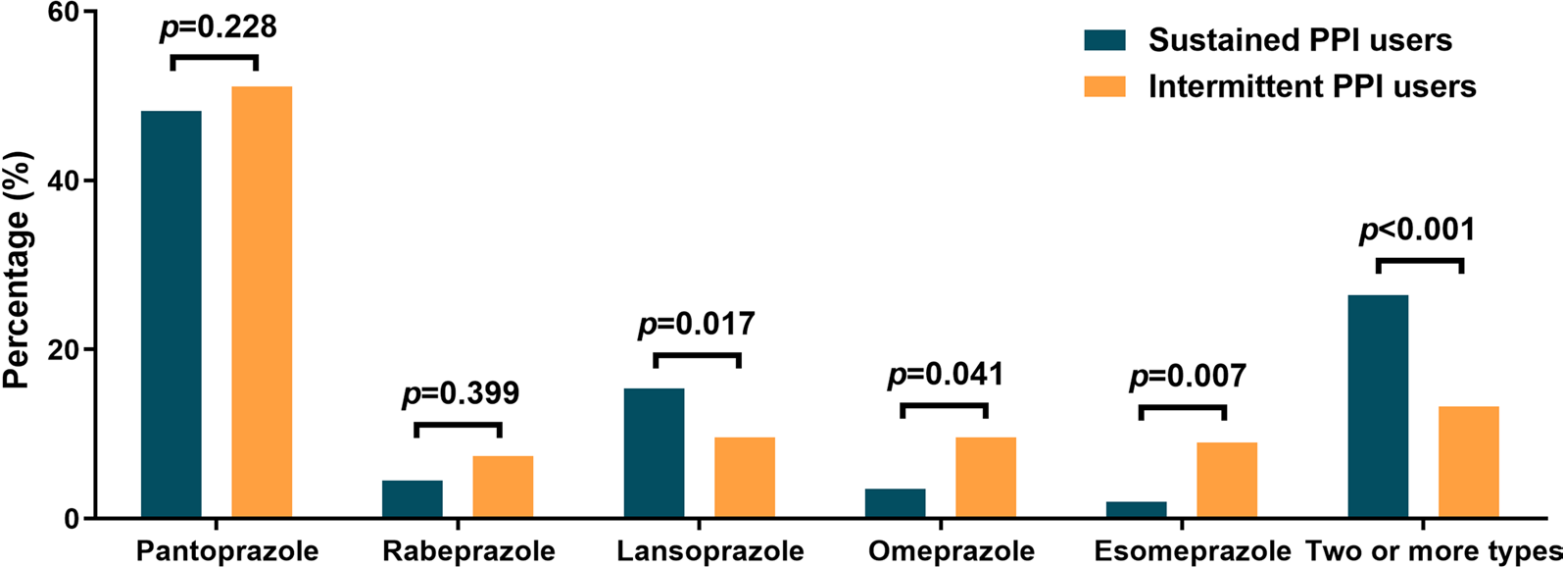
Comparison of different types of proton pump inhibitors between sustained and intermittent user group. PPI, proton pump inhibitor

A total of 1151 patients with CHD were included. After excluding 513 patients who did not meet the inclusion criteria, the final study cohort consisted of 638 patients (Fig. 1). According to the duration of PPIs treatment, we divided the patients into three groups: 201 were sustained PPI users, 188 were intermittent PPI users, and the remaining 249 were non-PPI users. Tables 1, 2 and 3 summarized the baseline characteristics, medical history and laboratory parameters of enrolled patients among three groups, respectively. Patients with sustained PPI use suffered with the highest rate of MI but the lowest level of BMI, the least rate of DM; while those without PPI use exhibited the highest rate of stroke (Table 1). Patients with sustained PPI use suffered more gastroduodenal ulcer than the other two groups (Table 1). Particularly, although sustained PPI users tended to have higher rate of aspirin treatment than the other two, no significant differences were found among these three groups (Table 2). Among the patients with PPIs treatment, about 50.0% used pantoprazole, 12.6% used lansoprazole, and about 20.1% patients used two or more types of PPIs during the follow-up period (Table 2). The percentages of lansoprazole, omeprazole and esomeprazole treatment were significantly different between sustained and intermittent PPI users (Table 2; Fig. 2). Moreover, sustained PPI users tended to use two or more types of PPIs than the intermittent ones (Fig. 2). There were no differences among three groups in other variables such as age, sex, blood pressure, smoking state (Table 1), CYP2C19 genotypes or the laboratory indexes (Table 3).

**Table 1:** Baseline characteristics of patients with sustained, intermittent and non-PPI users

	Sustained PPI users	Intermittent PPI users	Non-PPI users	*p* value
N	201	188	249	
Demographic data				
Age (y)	66.9 ± 11.1	64.4 ± 11.4	65.2 ± 10.7	0.080
Male (n, %)	123 (61.2%)	116 (61.7%)	174 (69.9%)	0.093
BMI (kg/m^2^)	23.75 ± 4.30	24.17 ± 3.86	24.76 ± 2.97	0.022
SBP (mmHg)	132.9 ± 20.8	134.8 ± 23.3	136.7 ± 21.1	0.180
DBP (mmHg)	75.4 ± 12.4	77.1 ± 13.3	76.5 ± 12.3	0.408
Smoking				0.880
Current	58 (28.9%)	60 (31.9%)	71 (28.5%)	
Former	31 (15.4%)	24 (12.8%)	39 (15.7%)	
None	112 (55.7%)	104 (55.3%)	139 (55.8%)	
Alcohol				0.037
Current	16 (8.0%)	15 (8.0%)	7 (2.8%)	
Former	5 (2.5%)	10 (5.3%)	15 (6.0%)	
None	180 (89.6%)	163 (86.7%)	227 (91.2%)	
Comorbidity				
CHD	82 (40.8%)	57 (30.3%)	94 (37.8%)	0.088
MI	35 (17.4%)	16 (8.5%)	35 (14.1%)	0.035
CHF	10 (5.0%)	10 (5.3%)	11 (4.4%)	0.906
CHD family history	26 (12.9%)	15 (8.0%)	22 (8.8%)	0.204
Hypertension	144 (71.6%)	128 (68.1%)	179 (71.9%)	0.645
Hypercholesterolemia	39 (19.4%)	34 (18.1%)	53 (21.3%)	0.700
Atrial fibrosis	15 (7.5%)	11 (5.9%)	7 (2.8%)	0.076
TIA	1 (0.5%)	2 (1.1%)	1 (0.4%)	0.659
Stroke	34 (16.9%)	19 (10.1%)	47 (18.9%)	0.037
DM	43 (21.4%)	61 (32.4%)	80 (32.1%)	0.019
CKD	12 (6.0%)	17 (9.0%)	16 (6.4%)	0.440
Gastroduodenal ulcer	45 (22.4%)	28 (14.9%)	20 (8.0%)	< 0.001
GI bleeding	4 (2.0%)	2 (1.1%)	4 (1.6%)	0.762
Surgical history				
PCI	50 (24.9%)	39 (20.7%)	70 (28.1%)	0.211
CABG	0 (0.0%)	1 (0.5%)	2 (0.8%)	0.460
Device implantation	6 (3.0%)	0 (0.0%)	7 (2.8%)	0.062
Other surgical history	32 (15.9%)	30 (16.0%)	45 (18.1%)	0.781

BMI, body mass index; CABG, coronary artery bypass graft; CHD, coronary heart disease; CHF, chronic heart failure; CKD, chronic kidney disease; DBP, diastolic blood pressure; DM, diabetes mellitus; GI, gastrointestinal; MI, myocardial infarction; PCI, percutaneous coronary intervention; PPI, proton pump inhibitor; SBP, systolic blood pressure; TIA, transient ischemic attack

**Table 2:** Medical history of patients with sustained, intermittent and non-PPI users

	Sustained PPI users	Intermittent PPI users	Non-PPI users	*p* value
N	201	188	249	
Medicine history				
Aspirin	149 (74.1%)	125 (66.5%)	170 (68.3%)	0.221
PPI				
Pantoprazole	97 (48.2%)	96 (51.1%)	–	0.228
Rabeprazole	9 (4.5%)	14 (7.4%)	–	0.399
Lansoprazole	31 (15.4%)	18 (9.6%)	–	0.017
Omeprazole	7 (3.5%)	18 (9.6%)	–	0.041
Esomeprazole	4 (2.0%)	17 (9.0%)	–	0.007
Two or more types	53 (26.4%)	25 (13.3%)	–	< 0.001
ACEI/ARB	140 (69.7%)	120 (63.8%)	152 (61.0%)	0.160
CCB	98 (48.8%)	78 (41.5%)	112 (45.0%)	0.354
β blocker	162 (80.6%)	133 (70.7%)	174 (69.9%)	0.022
Statin	192 (95.5%)	174 (92.6%)	223 (89.6%)	0.061
Warfarin	4 (2.0%)	1 (0.5%)	1 (0.4%)	0.175

ACEI, angiotensin converting enzyme inhibitor; ARB, angiotensin II receptor antagonist; CCB, calcium channel blocker; PPI, proton pump inhibitor

**Table 3:** Laboratory parameters of patients with sustained, intermittent and non-PPI users

	Sustained PPI users	Intermittent PPI users	Non-PPI users	*p* value
N	201	188	249	
*Laboratory parameters*				
Creatinine (mg/dL)	104.5 ± 70.8	111.3 ± 97.8	103.2 ± 56.7	0.552
Glucose (mmol/L)	5.5 ± 1.8	5.9 ± 2.4	5.7 ± 1.9	0.171
Total cholesterol (mmol/L)	4.22 (3.51, 5.11)	4.48 (3.81, 5.42)	4.47 (3.65, 5.25)	0.597
HDL-C (mmol/L)	1.07 (0.90, 1.32)	1.08 (0.89, 1.29)	1.06 (0.90, 1.26)	0.585
LDL-C (mmol/L)	2.46 (1.98, 3.12)	2.64 (2.14, 3.29)	2.59 (2.08, 3.19)	0.723
Triglycerides (mmol/L)	1.20 (0.94, 1.86)	1.38 (0.94, 2.04)	1.45 (1.05, 2.09)	0.239
Uric acid (µmol/L)	368.0 (311.0, 446.0)	393.0 (319.0, 477.0)	391.0 (329.0, 469.5)	0.916
ALT (U/L)	21.0 (15.0, 33.0)	20.0 (14.0, 35.5)	21.0 (15.0, 32.0)	0.455
AST (U/T)	23.0 (18.0, 38.0)	21.0 (18.0, 34.5)	23.0 (18.0, 29.0)	0.954
CK (U/L)	94.0 (64.0, 162.0)	87.0 (62.5, 136.0)	90.5 (65.0, 168.5)	0.758
CK-MB (U/L)	14.0 (11.0, 20.0)	14.0 (11.0, 19.0)	13.0 (11.0, 17.0)	0.567
hs-CRP (mg/L)	7156.0 (5888.0, 8098.0)	7439.0 (6282.0, 8259.0)	7544.5 (6269.8, 8507.0)	0.228
HbAlc (%)	6.0 (5.6, 6.6)	6.0 (5.6, 7.1)	6.1 (5.6, 7.1)	0.580
NT-proBNP	126.3 (55.1, 674.7)	151.0 (66.4, 587.6)	159.9 (63.2, 697.5)	0.732
TnT-HS	7.4 (4.4, 50.7)	13.2 (5.0, 398.0)	11.1 (5.4, 67.3)	0.439
Hb (g/L)	131.5 ± 19.1	129.4 ± 18.7	134.7 ± 22.5	0.042
Platelet count (× 10^9^/L)	221.6 ± 60.8	235.5 ± 64.3	221.9 ± 62.2	0.059
CYP2C19 genotypes (%)	110	111	115	0.536
EM	31.8	39.6	42.6	
IM	50.0	45.0	40.9	
PM	18.2	15.3	16.5	

ALT, alanine transaminase; AST, aspartate aminotransferase; CK, creatine kinase; CK-MB, creatine kinase isoenzymes; EM, extensive metabolizers; Hb, hemoglobin; HbAlc, glycated hemoglobin; HDL-C, high-density lipoprotein cholesterol; hs-CRP, hyper-sensitive C-reactive protein; IM, intermediate metabolizers; LDL-C, low-density lipoprotein cholesterol; NT-proBNP, N-terminal (NT)-pro hormone BNP; PM, poor metabolizers; TNT-HS, high sensitive troponin T

### Effect of PPIs use on clinical outcomes after 18-month follow-up

**Fig. 3:**
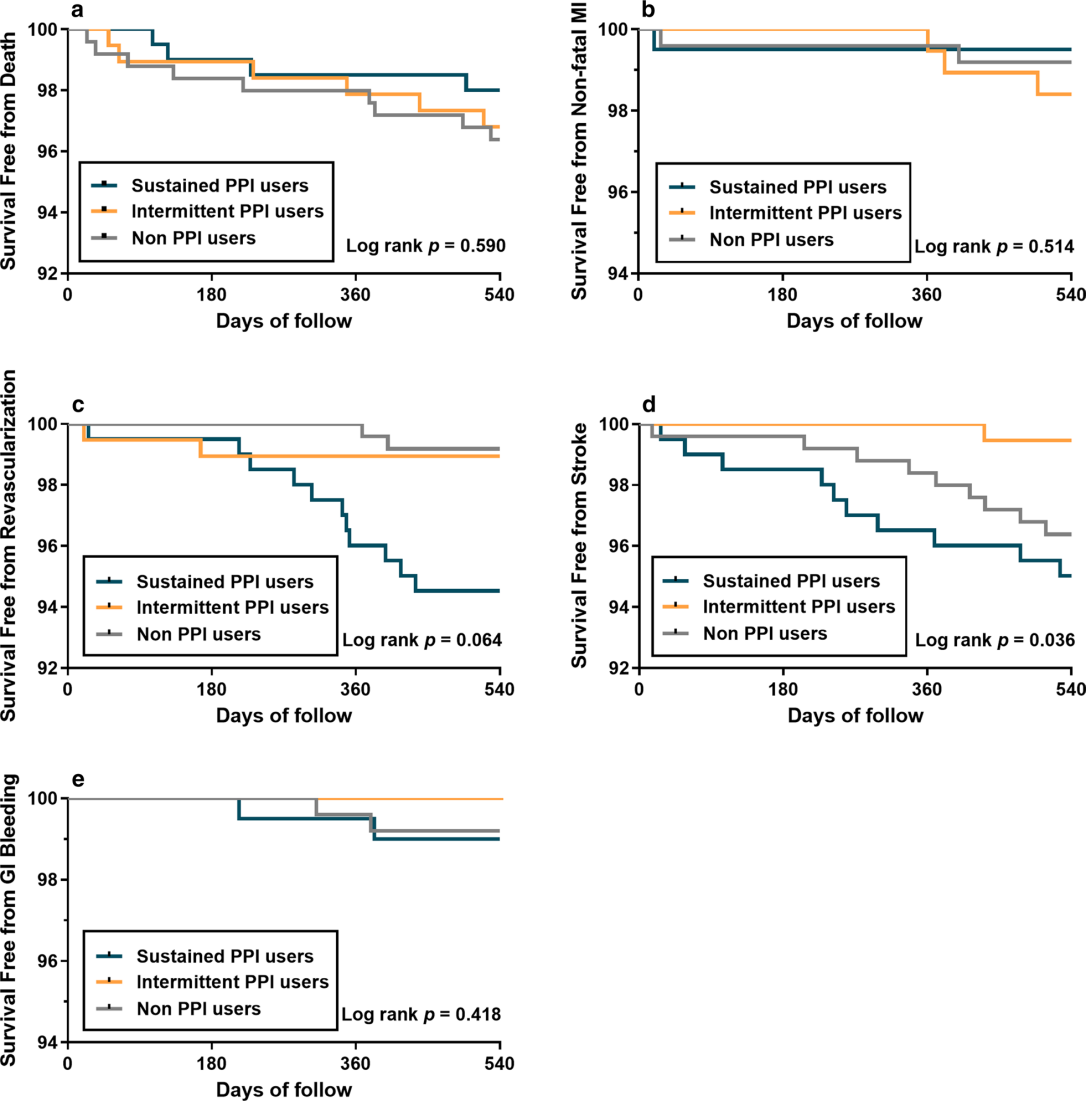
Kaplan–Meier survival curves representing the estimated cumulative incidence of all-caused death (**a**), non-fatal MI (**b**), revascularization (**c**), stroke (**d**), and GI bleeding (**e**) after 18 months in sustained, intermittent users and non-users groups. GI, gastrointestinal; MI, myocardial infarction; PPI, proton pump inhibitor

**Fig. 4:**
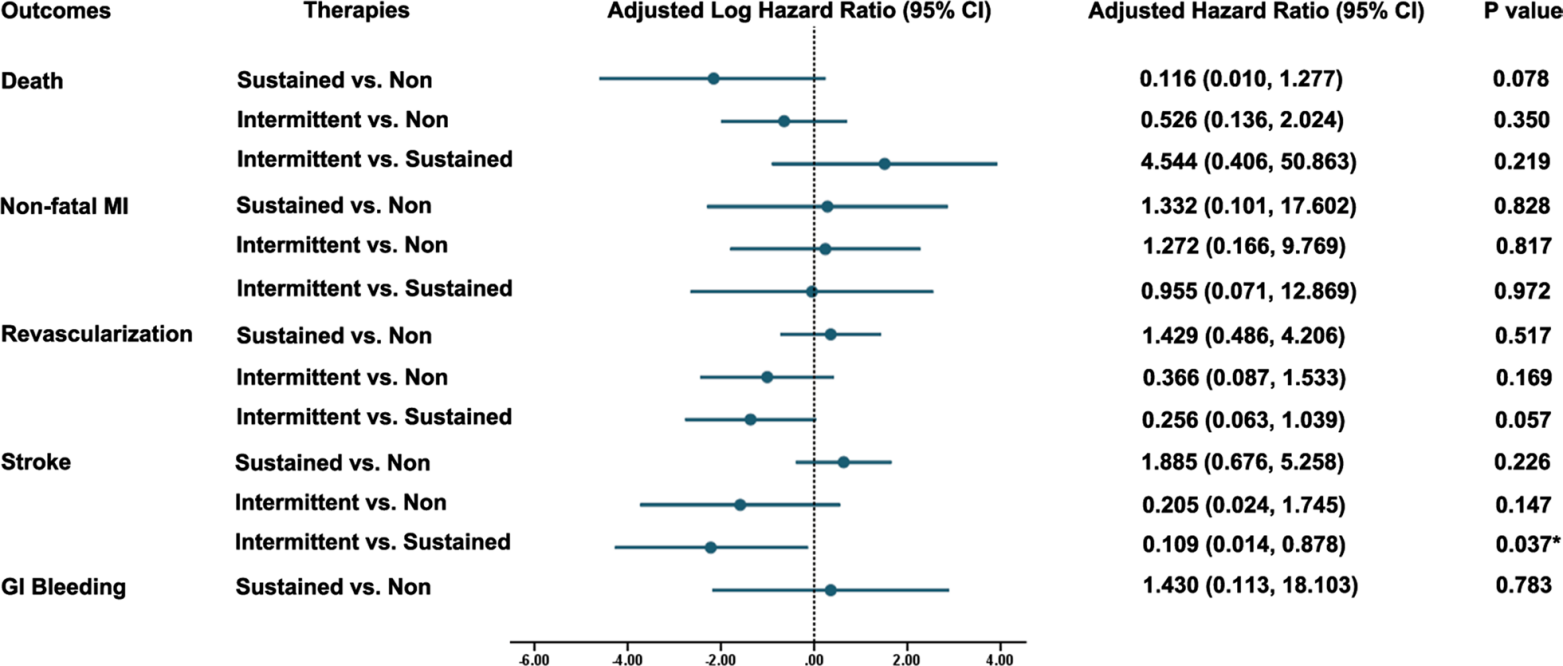
Multivariate COX proportional hazard regression analysis of clinical events in sustained PPI, intermittent PPI and non-PPI users. GI, gastrointestinal; MI, myocardial infarction; PPI, proton pump inhibitor

As shown in Table 4, patients with sustained PPIs use had the highest rate of 18-month stroke and re-hospitalization, mainly complaint of angina (Table 4). Stroke incidence was also the highest in sustained PPI users (Table 4). The Kaplan–Meier analysis revealed that PPIs use was significantly associated with 18-month rate of stoke (log rank *p* = 0.036; Fig. 3d). Further multivariable Cox regression analysis confirmed that intermittent use of PPIs was associated with lower risk of stroke compared with the sustained ones (adjusted HR: 0.109, 95% CI 0.014–0.878; *p* = 0.037) but not non-PPI ones (adjusted HR: 0.205, 95% CI 0.024–1.745, *p* = 0.147; Fig. 4). However, no significant effects of PPIs use were observed on the all-cause mortality, non-fatal MI or revascularization (Figs. 3a–c, 4). Particularly, GI bleeding was also not significantly different among these groups regardless of the PPIs use (Table 4, Fig. 3e).

**Table 4:** Clinical outcomes after 18-month follow-up in sustained PPI, intermittent PPI and non-PPI users

	Sustained PPI users	Intermittent PPI users	Non-PPI users	*p* value
Death	4 (2.0%)	6 (3.2%)	9 (3.6%)	0.589
Fatal MI	0 (0.5%)	1 (0.5%)	1 (0.4%)	0.612
Stroke	1 (0.5%)	1 (0.5%)	1 (0.4%)	0.978
Other cardiovascular	1 (0.5%)	1 (0.5%)	1 (0.4%)	0.978
Non-cardiovascular	0 (0.5%)	0 (0.0%)	4 (1.6%)	0.043
Unknown	2 (1.0%)	3 (1.6%)	2 (0.8%)	0.723
Rehospitalization	101 (50.2%)	89 (47.3%)	80 (32.1%)	< 0.001
MI	0 (0.0%)	3 (1.6%)	3 (1.2%)	0.227
Angina	46 (22.9%)	26 (13.8%)	29 (11.6%)	0.003
Stroke	3 (1.5%)	1 (0.5%)	5 (2.0%)	0.429
GI bleeding	1 (0.5%)	0 (0.0%)	0 (0.0%)	0.337
Other	51 (25.3%)	59 (47.9%)	43 (17.3%)	0.002
Cardiovascular events				
Non-fatal MI	1 (0.5%)	3 (1.6%)	2 (0.8%)	0.512
Revascularization	11 (5.5%)	3 (1.6%)	6 (2.4%)	0.063
Stroke	10 (5.0%)	1 (0.5%)	9 (3.6%)	0.036
GI bleeding	2 (1.0%)	0 (0.0%)	2 (0.8%)	0.417
Other bleeding	0 (0.0%)	0 (0.0%)	0 (0.0%)	–

GI, gastrointestinal; MI, myocardial infarction; PPI, proton pump inhibitor

### The association between PPIs use and 18-month MACE

**Fig. 5:**
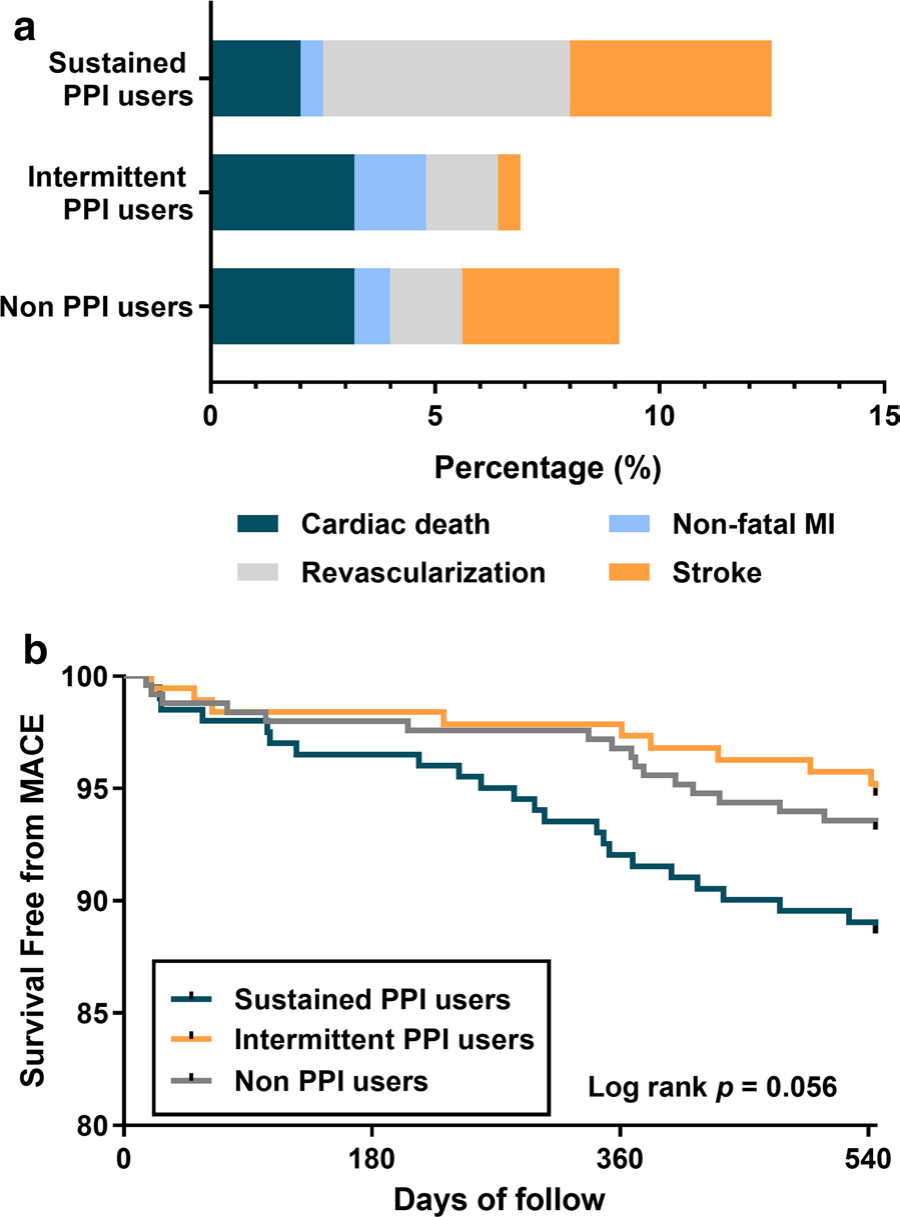
Comparison of the risk of MACE after 18 months among sustained PPI, intermittent PPI and non-PPI users. **a** Proportion of MACE after 18 months in sustained PPI, intermittent PPI and non-PPI users. **b** Kaplan–Meier survival curves representing the estimated cumulative incidence of MACE after 18 months in sustained PPI, intermittent PPI and non-PPI users. MI, myocardial infarction; MACE, Major adverse cardiac events; PPI, proton pump inhibitor

Beside analyzing the association between PPI use and the individual clinical outcome, we also compared the rate of MACE among three groups. Patients with intermittent PPI use tended to suffer the lowest rate of MACE while sustained users had the highest rate (6.8% for non-PPI users, 5.3% for intermittent users, and 11.4% for sustained ones) (Fig. 5a) but the tendency was nonsignificant (log rank *p* = 0.056, Fig. 5b). In multivariable Cox regression analysis, intermittent use of PPI was associated with lower risk of MACE than the sustained PPI users (sustained PPI users vs. non-PPI users: adjusted HR: 1.678, 95% CI 0.822–3.423; *p* = 0.155; intermittent PPI users vs. non-PPI users: adjusted HR: 0.492, 95% CI 0.194–1.248; *p* = 0.135; intermittent PPI users vs. sustained PPI users: adjusted HR: 0.293, 95% CI 0.119–0.722; *p* = 0.008).

### The association between PPIs use and 18-month NACE

**Fig. 6:**
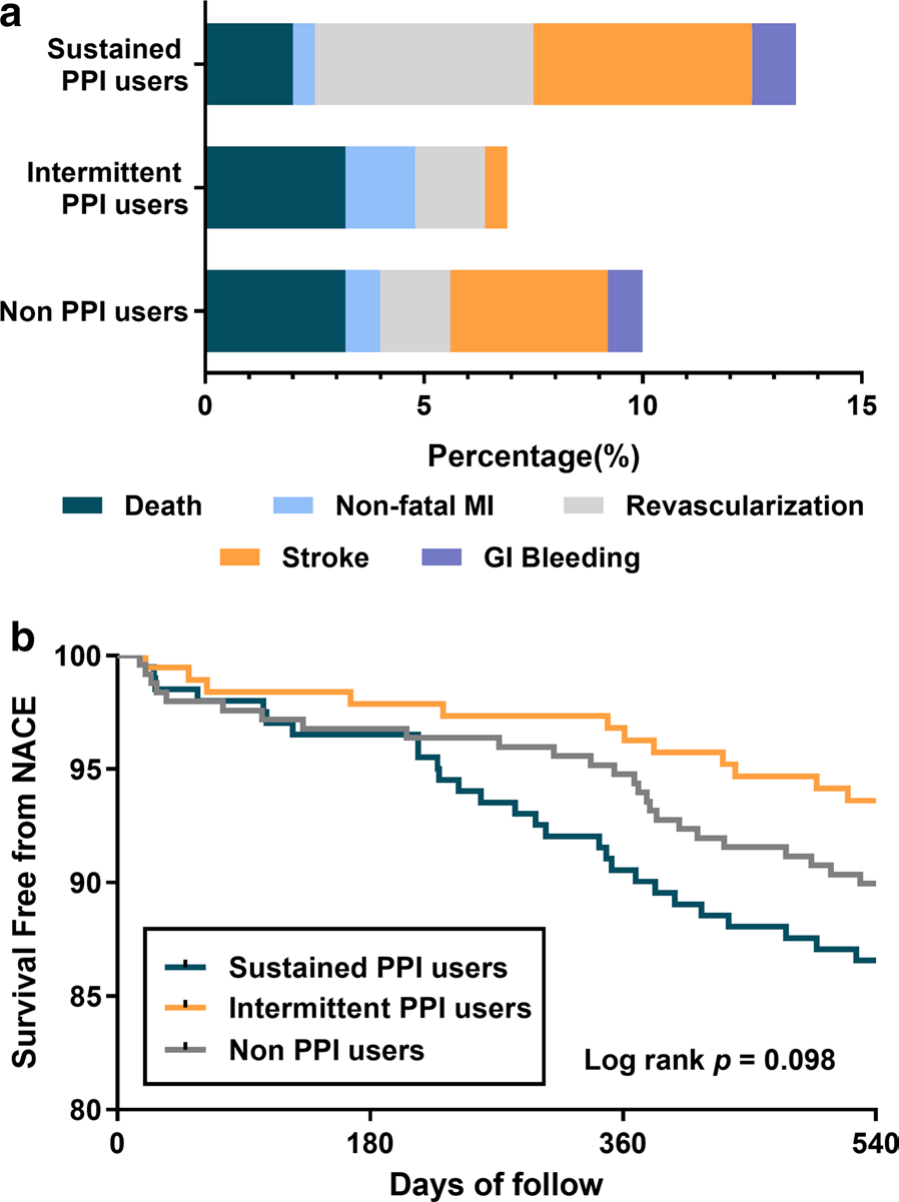
Comparison of the risk of NACE after 18 months among sustained PPI, intermittent PPI and non-PPI users. **a** Proportion of NACE after 18 months in sustained PPI, intermittent PPI and non-PPI users. **b** Kaplan–Meier survival curves representing the estimated cumulative incidence of NACE after 18 months in sustained PPI, intermittent PPI and non-PPI users. GI, gastrointestinal; MI, myocardial infarction; NACE, net adverse clinical events; PPI, proton pump inhibitor

In addition, NACE, including all adverse events, were also estimated. The rates of NACE among three groups were nonsignificant (log rank *p* = 0.098, Fig. 6). Multivariable Cox regression analysis further showed that intermittent use of PPIs was associated with lower risk of NACE compared with the sustained PPI users, but not non-PPI users (Table 5). To further explore the possible risk factors affecting NACE, subgroup analysis was conducted. As shown in Table 5, intermittent use of PPIs was associated with lower risk of NACE than the other two groups for patients with PCI. Besides, intermittent PPI male users with age over 60 years old, hypertension, CKD and CYP2C19 IM type also suffered less risk of NACE compared with sustained PPI users (Table 5). No different risk ratios of NACE were detected in the subgroups of patients with or without gastroduodenal ulcer (*p* > 0.05; Table 5). Neither types of the PPIs including pantoprazole exerted beneficial effects on MACE or NACE (Table 5 and Additional file 1: Table S1).

**Table 5:** Subgroup analysis of NACE in sustained PPI, intermittent PPI users and non-PPI users

Subgroup	No. of events (%)			Adjusted hazard ratio (95% confidence interval), *p* value		
	Sustained PPI users	Intermittent PPI users	Non-PPI users	Sustained versus non	Intermittent versus non	Intermittent versus sustained
Overall	27 (13.4)	13 (6.9)	25 (10.0)	1.309 (0.687, 2.495), *p* = 0.413	0.467 (0.213, 1.025), *p* = 0.058	0.357 (0.162, 0.786), *p* = 0.011
Age						
≤ 60 y	3 (5.2)	4 (5.1)	6 (7.3)	1.152 (0.128, 10.388), *p* = 0.899	0.534 (0.055, 5.155), *p* = 0.588	0.464 (0.039, 5.486), *p* = 0.542
> 60 y	24 (16.8)	9 (8.2)	19 (11.4)	1.392 (0.692, 2.802), *p* = 0.353	0.440 (0.170, 1.138), *p* = 0.090	0.316 (0.122, 0.818), *p* = 0.018
Gender						
Male	21 (17.1)	8 (6.9)	13 (7.5)	2.079 (0.937, 4.611), *p* = 0.072	0.526 (0.193, 1.434), *p* = 0.209	0.253 (0.097, 0.664), *p* = 0.005
Female	6 (7.7)	5 (6.9)	12 (16.0)	0.411 (0.113, 1.489), *p* = 0.176	0.380 (0.097, 1.496), *p* = 0.166	0.925 (0.198, 4.317), *p* = 0.921
Hypertension						
Yes	23 (16.0)	9 (7.0)	20 (11.2)	1.234 (0.617, 2.466), *p* = 0.552	0.360 (0.135, 0.964), *p* = 0.042*	0.292 (0.109, 0.785), *p* = 0.015
No	4 (7.0)	4 (6.7)	5 (7.1)	0.958 (0.140, 6.574), *p* = 0.965	0.328 (0.040, 2.725), *p* = 0.302	0.343 (0.038, 3.079), *p* = 0.339
Stroke						
Yes	7 (20.6)	2 (10.5)	12 (25.5)	0.668 (0.206, 2.164), *p* = 0.501	0.306 (0.031, 3.025), *p* = 0.311	0.457 (0.047, 4.469), *p* = 0.501
No	20 (12.0)	11 (6.5)	13 (6.4)	2.433 (1.020, 5.805), *p* = 0.045	0.649 (0.258, 1.631), *p* = 0.358	0.267 (0.104, 0.686), *p* = 0.066
DM						
Yes	8 (18.6)	8 (13.1)	11 (13.8)	2.080 (0.647, 6.692), *p* = 0.219	0.707 (0.233, 2.151), *p* = 0.542	0.340 (0.092, 1.257), *p* = 0.106
No	19 (12.0)	5 (3.9)	14 (8.3)	1.017 (0.465, 2.224), *p* = 0.966	0.369 (0.115, 1.185), *p* = 0.094	0.362 (0.118, 1.117), *p* = 0.077
CKD						
Yes	25 (15.9)	12 (8.1)	25 (14.2)	1.220 (0.633, 2.351), *p* = 0.552	0.441 (0.195, 0.999), *p* = 0.050	0.361 (0.157, 0.833), *p* = 0.017
No	2 (4.5)	1 (2.5)	0 (0.0)	–	–	–
Gastroduodenal ulcer						
Yes	4 (9.1)	1 (3.6)	5 (26.3)	0.485 (0.048, 4.857), *p* = 0.538	0.191 (0.014, 2.550), *p* = 0.210	0.393 (0.034, 4.527), *p* = 0.454
No	23 (14.7)	12 (7.5)	20 (8.7)	1.740 (0.856, 3.537), *p* = 0.126	0.583 (0.248, 1.369), *p* = 0.215	0.335 (0.142, 0.789), *p* = 0.012
PPI types						
Pantoprazole	24 (16.8)	8 (7.2)	25 (10.0)	1.176 (0.508, 2.718), *p* = 0.705	0.505 (0.196, 1.303), *p* = 0.158	0.430 (0.150, 1.230), *p* = 0.115
Others	3 (5.2)	5 (6.4)	25 (10.0)	1.127 (0.514, 2.471), *p* = 0.766	0.381 (0.121, 1.202), *p* = 0.100	0.338 (0.098, 1.169), *p* = 0.087
PCI						
Yes	18 (11.9)	3 (2.5)	14 (9.3)	1.166 (0.522, 2.602), *p* = 0.708	0.194 (0.052, 0.731), *p* = 0.015	0.167 (0.045, 0.617), *p* = 0.007
No	9 (18.0)	10 (14.7)	11 (11.1)	1.595 (0.532, 4.783), *p* = 0.405	0.954 (0.341, 2.671), *p* = 0.929	0.598 (0.178, 2.014), *p* = 0.407
CYP2C19 genotypes						
EM	5 (14.3)	2 (4.5)	5 (10.2)	4.173 (0.606, 28.750), *p* = 0.147	0.624 (0.088, 4.402), *p* = 0.636	0.149 (0.022, 1.031), *p* = 0.054
IM	9 (16.4)	4 (8.0)	6 (12.8)	3.652 (0.916, 14.559), *p* = 0.066	0.664 (0.156, 2.827), *p* = 0.580	0.182 (0.044, 0.751), *p* = 0.019
PM	2 (3.6)	1 (6.3)	2 (10.5)	–	0.058 (0.002, 1.887), *p* = 0.109	–

CKD, chronic kidney disease; DM, diabetes mellitus; EM, extensive metabolizers; GI, gastrointestinal; IM, intermediate metabolizers; MI, myocardial infarction; NACE, net adverse clinical events; PCI, percutaneous coronary intervention; PM, poor metabolizers; PPI, proton pump inhibitor

## Discussion

The results of this study suggest that in patients with CHD taking clopidogrel, intermittent use of PPIs did not increase the risk of all-cause death, cardiovascular adverse events, and GI bleeding after 18-month follow-up. Instead, compared with the sustained PPI users, intermittent use of PPIs was associated with a reduced risk of NACE after 18 months, especially for male CHD patients with an age over 60 years old after PCI, with hypertension or CKD.

Clopidogrel, often combined with aspirin, is a gold standard treatment for CHD, especially for patients after PCI, to reduce the incidence of cardiovascular adverse events [18–20]. However, since clopidogrel needs to be metabolized by the liver CYP450 enzyme system, combined use of the drug metabolized by this enzyme may exert inhibitory effect on clopidogrel [21]. One of the most concerned medications is PPIs, which are often used to prevent GI bleeding [22]. Previous pharmacodynamic studies have shown that the platelet inhibitory rate decreased when clopidogrel was combined with PPIs [23–26]. Most observational studies found that patients under both clopidogrel and PPIs therapy had an increased risk of ischemic cardiovascular events [4, 9, 10, 27–29]. In the BASKET study, the results of subgroup analysis showed that patients who took PPIs had a higher incidence of NACEs and MI after 3 years than those who did not [10]. The following CAPRIE study confirmed that patients with PPIs suffered a higher risk of ischemic cardiovascular events after one year than non-PPI users [9]. The results of the ADAPT-DES study further proved that the combination of PPIs was associated with increased platelet resistance, and that the incidence of NACE was significantly increased after 2 years [4]. These results indicated that PPIs were able to affect its antiplatelet activity of clopidogrel by disturbing its metabolism, and thereby led to an increased risk of cardiovascular adverse events. However, the results of randomized controlled trials (RCTs) and biased clinical studies were inconsistent [7, 11, 30, 31]. Results from PRINCIPLE-TIMI 44 and TRITON-TIMI 38 studies suggested that clopidogrel and PPIs use had no effects on the clinical adverse events, although the platelet activity induced by clopidogrel was affected by the concurrent use of PPIs [7]. The subsequent COGENT study included a multicenter randomized double-blind case–control study involving 5000 patients with ACS or stent implantation and randomly assigned to omeprazole based on DAPT. After 180 days of follow-up, studies confirmed that the incidence of total cardiovascular death, MI, target vessel revascularization and stroke was not significantly different in both groups when compared to placebo group [12]. Another recent RCT, the PRODIGY study, showed the similar results [11]. Studies in Asian populations have found that clopidogrel combined with PPIs was not associated with cardiovascular events (such as AMI, cardiovascular death, etc.) [32, 33]. The above series of clinical research conclusions were consistent with the results of this study. In this study, compared with patients without use of PPIs, intermittent or sustained use of PPIs did not significantly increase the occurrence of all-cause deaths and adverse clinical events. This further confirmed that although the combined use of PPIs might reduce the antiplatelet function caused by clopidogrel, it did not necessarily indicate an increased risk of clinical adverse events after combined use [34, 35]. Indeed, a recent clinical study involving more than 60,000 patients with gastroesophageal reflux disease suggested that patients taking PPIs were 1.2 times more likely to develop MI than non-PPI users, and their risk of cardiovascular death was twice higher than those who did not take it, but all these were independent of clopidogrel use [29]. The latest meta-analysis of this issue did not find a definitive answer either [34, 35]. Therefore, the clinical prognosis of CHD patients with combined use of clopidogrel and PPIs still requires further confirmation by the well-designed RCTs.

The purpose of using PPIs is mainly to prevent or reduce the occurrence of GI bleeding. Although relatively few studies focused on the benefit of clopidogrel combined with PPIs on GI bleeding, the risk of GI bleeding was found to be reduced after combined with PPIs [11, 12, 31, 36]. In the COGENT study, although there was no significant difference in the overall incidence of cardiovascular adverse events, the risk of GI bleeding was lower in the omeprazole group than in the placebo after 180 days. However, this study was terminated early and the follow-up time was short. Therefore, the risk of GI bleeding after long-term treatment could not be evaluated in this study [12, 31]. The PRODIGY study found that the occurrences of BARC bleeding standard type 2, 3, and 5 bleeding events at 6 months and 24 months were not statistically different between groups with or without PPIs use. However, no comparison among various bleeding events such as GI bleeding events was conducted in that study [11]. This study focused on the observation of GI bleeding in patients with CHD taking clopidogrel combined with PPIs. There was no significant difference in GI bleeding incidence. However, among the sustained PPI users, 45 people suffered gastrointestinal ulcer disease, and the incidence of GI bleeding was 4.4% after 18 months; among the non-PPI users, 20 people suffered stomach intestinal ulcer disease, the incidence of GI bleeding was up to 10% after 18 months. It seemed that the occurrence of GI bleeding in the non-PPI users was higher than that in the sustained PPI users. Due to the small number of events, it could not be confirmed by multivariate analysis. However, this result was consistent with the research published earlier by the Chitose T. team in Japan, which suggested that the group without the PPIs use showed a tendency of increasing GI bleeding [36]. Therefore, use of PPIs was unlikely to increase the risk of clinical adverse events but may exert benefit on GI bleeding prevention.

How to prescribe PPIs to the CHD patients in clinical practice is another important issue. Till now, only few studies have explored the impact of various frequencies of PPIs use on clinical prognosis. Such as the PRODIGY study, the study defined those who took PPIs at the 30-day follow-up as the PPIs use group. Patients who took less than 30-day PPIs and those who discontinued PPIs were excluded [11]. Other studies, such as ADAPT-DES and BASKET, regarded patients discharged with PPIs as the PPI group, but did not consider the changes of PPIs dosage or the frequency of concurrent use after discharge [4, 10]. In the clinical practice, most patients may accept PPIs treatment when they suffer digestive discomfort. Therefore, to identify the optimal PPI regimens in CHD patients to achieve beneficial clinical impacts is of great importance. Based on the duration of PPI use during the follow-up period, PPI users were divided into the sustained and intermittent PPI users groups. No significant difference was found on the individual clinical adverse events of the two PPI-user groups. Surprisingly, the intermittent PPI user group had a significantly lower incidence of NACE than the sustained PPI users, of which the main benefit may be due to a reduction in stroke. Further subgroup analysis suggested that male CHD patients with an age over 60 old years, after PCI treatment, with hypertension or CKD, might benefit more from intermittent use of PPIs. Therefore, for certain populations, intermittent use may be related to a reduction in NACE. In fact, this was in line with the recommendations of the latest US ACC/AHA guidelines. The guideline considered that PPIs may be used appropriately for people at high risk, such as the elderly, who were at high risk for GI bleeding [19, 20]. In addition, for PCI patients, who were necessary to take DAPT continuously for at least 1 year, it was particularly critical to prevent complications such as GI bleeding that forced to discontinue DAPT. The present study indicated that the appropriate and intermittent concurrent use of PPI with clopidogrel may be available for patients after PCI. Nevertheless, because of the low incidence of GI bleeding and other bleeding events in this study, it was impossible to further analyze whether the benefit of NACE came from the reduction of GI bleeding or was affected by the imbalanced baseline. However, multivariate analysis adjusted for possible confounders reduced the impact of baseline imbalances to a certain extent. According to previous large-scale clinical studies such as the PRODIGY study, the incidence of NACE in the sustained user and the non-PPI user group in this study was comparable, also between 10.0 and 15.0%, while the intermittent user group was significantly reduced, which indicated that the results of this study had considerable credibility [11].

There were several limitations in this study. Firstly, this study was a single-center retrospective study. Population included is heterogeneous, considering patients in single and dual antiplatelet therapy. The included sample size was relatively small. Thus, the inherent bias would inevitably occur. Secondly, the small number of clinical adverse events might have a certain impact on the research results. Thirdly, the occurrence of clinical events and the consumption of drugs were obtained by telephone or outpatient follow-up, which might inevitably lead to reporting bias. However, the majority of baseline data, medication treatments, and some clinical adverse events were collected or confirmed in the hospital medical system that would minimize the reporting bias. Fourthly, there are only 65 NACEs in this study, while the multivariable model used 11 covariates, which may have a high risk of overfitting. Thus, we should be cautious when interpreting the current findings.

## Conclusion

In summary, the intermittent combination of clopidogrel and PPI did not increase the risk of all-cause death and cardiovascular adverse events in patients with CHD. Intermittent use of PPIs is associated with a lower rate of MACE and NACE. Although there was no statistically significant difference in the incidence of GI bleeding among the three groups, there was a tendency of decreased incidence of GI bleeding with the use of PPIs. However, larger RCTs are needed to further confirm the conclusions of this study.

## Supplementary Information

**Additional file 1.** **Supplementary Table S1.** Unadjusted Hazard Ratios for MACE and NACE according to Specific PPIs Compared with Non-PPI Users.

## Data Availability

The datasets used and analyzed during the current study are available from the corresponding author on reasonable request.

## Abbreviations

ACEI: Angiotensin converting enzyme inhibitor
ACS: Acute coronary syndrome
AF: Atrial fibrillation
ALT: Alanine transaminase
AMI: Acute myocardial infarction
ANOVA: Analysis of variance
ARB: Angiotensin II receptor antagonist
AST: Glutamic-oxaloacetic transaminase
BARC: Bleeding Academic Research Consortium
BMI: Body mass index
BP: Blood pressure
CCB: Calcium channel blocker
CHD: Coronary heart disease
CHF: Chronic heart failure
CI: Confidence interval
CKD: Chronic kidney disease
CK-MB: Creatine phosphokinase isoenzyme
CVD: Cardiovascular diseases
CYP: Cytochrome
DAPT: Dual antiplatelet therapy
DM: Diabetes mellitus
EMs: Extensive (normal) metabolizers
FPG: Fasting plasma glucose
GERD: Gastroesophageal reflux disease
GI: Gastrointestinal
GOF: Gain-of-function
HDL-C: High-density lipoprotein cholesterol
HR: Hazard ratios
hs-CRP: High-sensitivity C-reactive protein
IMs: Intermediate metabolizers
LDL-C: Low-density lipoprotein cholesterol
MACE: Major adverse cardiac event
MI: Myocardial infarction
NACE: Net adverse clinical event
NSAIDS: Non-steroidal anti-inflammatory drugs
PCI: Percutaneous coronary intervention
PMs: Poor metabolizers
PPIs: Proton pump inhibitors
RCTs: Randomized controlled trials
SD: Standard deviation
TG: Triglyceride
TIA: Transient ischemic attack
TC: Total cholesterol
UMs: Ultra-rapid metabolizers
