# Supplementary material for: Intermittent concurrent use of clopidogrel and proton pump inhibitors did not increase risk of adverse clinical outcomes in Chinese patients with coronary artery disease
Source: BMC Cardiovasc Disord. 2021 Feb 5;21:75. doi: 10.1186/s12872-021-01884-z (PMC7863361; doi:10.1186/s12872-021-01884-z)
Supplement: Supplementary file 1 — Additional file 1. Supplementary Table S1. Unadjusted Hazard Ratios for MACE and NACE according to Specific PPIs Compared with Non-PPI Users. [file 12872_2021_1884_MOESM1_ESM.docx]

Additional file 1: Table S1. Unadjusted Hazard Ratios for MACE and NACE according to Specific PPIs Compared with Non-PPI Users.

| PPIs | vs. Non-PPI Users | |
| --- | --- | --- |
|  | MACE | NACE |
| Pantoprazole (n=193) | 0.765 (0.391-1.499) | 0.960 (0.533-1.729) |
| Rabeprazole (n=23) | 1.579 (0.210-11.862) | 1.180 (0.279-4.982) |
| Lansoprazole (n=49) | 3.436 (0.457-25.820) | 5.144 (0.697-37.963) |
| Omeprazole (n=25) | 23.347 (0.018-30096.877) | 2.636 (0.357-19.453) |
| Esomeprazole (n=21) | 0.725 (0.168-3.140) | 1.091 (0.258-4.607) |

MACE, major adverse cardiac events; NACE, net adverse clinical events; PPIs, proton pump inhibitors.
